# Efficacy and safety of multimodal analgesic techniques for preventing chronic postsurgery pain under different surgical categories: a meta-analysis

**DOI:** 10.1038/s41598-017-00813-5

**Published:** 2017-04-06

**Authors:** Jun Zhou, Youling Fan, Jiying Zhong, Xianjie Wen, Hongtao Chen

**Affiliations:** 1 Department of Anesthesiology, First People’s Hospital of Foshan, Foshan, 528000 Guangdong Province China; 2 Department of Anesthesiology, Guangzhou Panyu Central Hopital of Panyu District, Guangzhou, 511400 Guangdong Province China; 3 Department of Anesthesiology, Eighth People’s Hospital of Guangzhou, Guangzhou, 510060 Guangdong Province China

## Abstract

The purpose of this meta-analysis was to compare the efficacy and safety of regional anesthesia to manage chronic postsurgery pain. A systematic search of PubMed, EmBase, and the Cochrane Central Register of Controlled Trials was performed to identify randomized controlled trials that focused on chronic pain frequency, analgesic consumption, and adverse effects under different surgical categories. We collected 21 trials assessing 1,980 patients for our meta-analysis. The summary of relative risks (RRs) and standard mean differences (SMDs) were calculated to measure the treatment effect of regional anesthesia. Results indicated that regional anesthesia significantly reduced the frequency of postsurgery pain (RR, 0.69; 95% confidence interval [CI], 0.56–0.85; p < 0.001). The results showed significant differences in overall patient satisfaction between applications with and without regional anesthesia (SMD, 1.95; 95%CI, 0.83–3.06; p = 0.001); however in other results, there were no significant differences between the two groups. Subgroup analysis suggested that regional anesthesia treatment might differ according to country. In conclusion, our study indicated that regional anesthesia was effective and safe in reducing the frequency of postsurgery pain and improved overall patient satisfaction; however, studies on the long-term efficacy and safety of regional anesthesia are still required to further confirm these findings.

## Introduction

Postsurgery pain is a major medial challenge for patients and clinical staff and results in physical discomfort, psychological harm, and hormonal disturbances^1^. The major causes for postsurgery pain are tissue injury, residual pneumoperitoneum, and phrenic neuropraxia^2^. The incidence of acute neuropathic pain in patients within days after surgery ranged from 1.0 to 3.0%, and acute postsurgery neuropathic pain persisting for ≥3.0 months was regarded as chronic^3, 4^. Patients undergoing various surgical procedures and those receiving adequate postsurgery analgesia should have a lower result on the visual analog scale (VAS) and better endocrine response, which, in turn, could accelerate wound recovery^5^. Further, postsurgery regional analgesia is widely used for pain control. It improves analgesic efficacy and reduces the need for opioids for pulmonary and gastrointestinal dysfunction and, thus, their side effects^5–7^.

Opioids are most commonly used to manage postsurgery pain, but are frequently associated with adverse effects, such as respiratory depression, drug addiction, and nausea and vomiting^8–10^; therefore, an additional effective analgesic approach must be found. Regional analgesia has been clearly shown to be effective in reducing postsurgery pain, and has been associated with less adverse effects than opioids^11–14^. In addition, regional anesthesia, such as epidurals, are used in procedures involving specific wound entry sites. Because of the advances in regional analgesia, it has been recommended as an alternative to opioids for controlling postsurgery pain. Previous meta-analyses studies evaluated the effects of regional analgesia on specific surgical sites^15–17^; however, comprehensive evaluation of the effects in preventing chronic postsurgery pain over that of traditional analgesics remains controversial.

Regional analgesia is effective in pain management and reduces the consumption of opioids, but a clear comparison of the differences in long-term pain control between regional and traditional analgesics is needed. Hence, in this study, we evaluated the efficacy and safety of regional analgesia in preventing postsurgery chronic pain. In addition, we compared the treatment effects of regional analgesia among patients after surgery who had different baseline characteristics.

## Results

### Literature search

**Figure 1:**
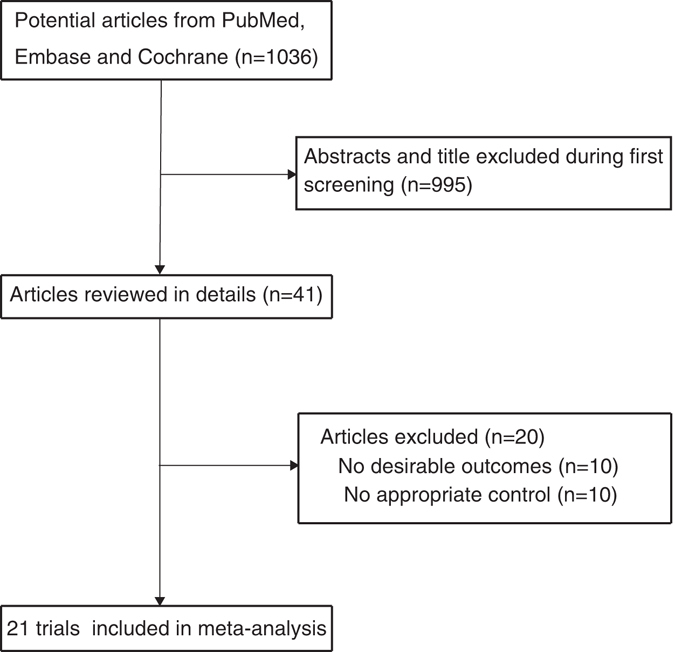
Study selection process.

The study retrieved 598 articles from PubMed, 942 from EmBase, and 263 from the Cochrane Library database and 1,036 articles were identified after removing the duplicates. Of these, 995 articles were excluded because of irrelevance after scanning the titles and abstracts. Additional full-text articles were reviewed and 20 more studies were excluded. Finally, 21 trials assessing 1,980 patients were collected for our systematic review^18–38^. The search process was showed in flow chart (Fig. 1).

### Study characteristics

The surgical type, including general, orthopedic, gynecological, and thoracotomy, were analyzed. Regional anesthesia methods included epidural, wound infusion, topical application, plexus blocks, spinal blocks, peritoneal instillation, and paravertebral blocks. The major effects measured were pain frequency, analgesia consumption, pain scale, and adverse effects. The follow-up duration ranged from 3.0 months to 4.7 y. According to Jadad scores, all studies were within the range of 1 to 4, so the overall quality of the included studies was not ideal (Table 1).

**Table 1:** Characteristics of subjects in eligible studies.

Author	Country	Sample size	Patients status	Regional technique	Outcomes	Follow-up duration	Jadad score
Katz^18^	Canada	83	Major Gynecologic Surgery by Laparotomy	Epidural	Pain frequency; Aggravating factors; Pain Disability Index; Mental Health Inventory; McGill; Worst pain since discharge;	6 months	2
Lavand’homme^19^	Belgium	80	Major Digestive Surgery	Epidural	Pain frequency; adverse events	12 months	3
Lavand’homme^20^	Belgium	92	Elective Cesarean Delivery	Continuous intrawound infusion	Pain frequency; Analgesic consumption	6 months	2
Karanikolas^21^	US	65	lower-limb amputation	Epidural	Pain frequency; McGilL	6 months	4
Singh^22^	US	26	posterior iliac crest bone graft harvesting	Wound irrigation	Pain frequency; VAS; functional activity score, overall satisfaction;	4.7 years	3
Fassoulaki^23^	Greece	50	Breast Surgery	Topical application	Pain frequency; No. of patients who needed analgesics	6 months	2
Senturk^24^	Turkey	69	Thoracotomy	Epidural	Pain frequency; Numerical Rating Scale	6 months	4
Bain^25^	Australia	40	Acromioplasty	Interscalene brachial plexus block	mean analgesic dosages	1 years	1
Burney^26^	US	34	Inguinal Hernia Repair	Spinal	Pain frequency	6 months	3
Shahin^27^	Egypt	370	Parietal Peritoneal Closure	Peritoneal instillation	Pain frequency; VAS	8 months	1
Bell^28^	Norway	8	breast-reduction surgery	Local infiltration	Pain frequency	6 months	2
Kairaluoma^29^	Finland	60	breast surgery	Single shot, paravertebral block	Pain frequency	1.0 year	3
Ju^30^	China	107	Thoracotomy	Epidural	Pain frequency	1.0 year	2
Paxton^31^	Ireland	70	Vasectomy	Local injection VAS deferens	Discomfort/no discomfort	1.0 year	1
Grosen^32^	Denmark	104	Thoracotomy	epidural infusion	Pain frequency; Pain Scale; analgesic consumption; adverse events	6 months	3
Strazisar^33^	Slovenia	60	breast carcinoma	local anaesthetic	Pain frequency	3 months	2
Kurmann^34^	Switzerland	357	inguinal hernia repair	local infiltration	Pain frequency	3 months	3
Chiu^35^	Canada	129	Breast Cancer Surgery	local anesthetic	Pain frequency	1.0 year	3
Suppa^36^	Italy	56	Cesarean section	Spinal anesthesia	Pain frequency; No. of patients who needed analgesics	3.0 years	2
Ilfeld^37^	US	60	Postmastectomy	single-injection thoracic paravertebral block	Pain frequency; Pain Scale	1.0 year	3
Zoric^38^	France	60	total hip arthroplasty	Single-shot intraoperative local anaesthetic infiltration	chronic pain level, analgesic consumption; adverse events	3.0 months	4

### Analysis results

**Figure 2:**
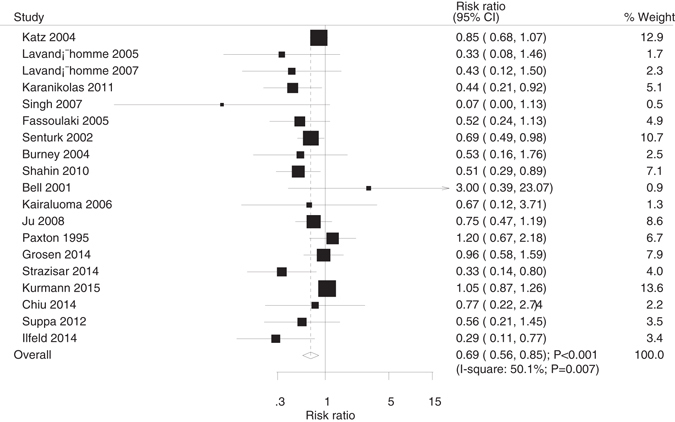
The effect of regional analgesia on chronic pain frequency.

The results reported that postsurgery pain frequency was significantly reduced in the 19 patients who underwent major surgery with regional anesthesia (RR, 0.69; 95%CI, 0.56–0.85; p < 0.001; Fig. 2); however, moderate heterogeneity was observed among the included studies (I^2^, 50.1%, p = 0.007). A sensitivity analysis showed that the results were not affected after sequentially excluding each trial. Subgroup analysis of pain frequency showed no significant differences between regional and traditional anesthesia in the trials conducted in Europe, sample size >100, and at surgery sites other than thoracotomy or laparotomy. In addition, RRR showed a statistically significant different effect of regional and traditional anesthesia on pain frequency in trials conducted in Europe when compared to trials conducted in other countries (Table 2).

**Table 2:** Subgroup analysis on pain frequency.

Variable	Subgroup	Number of trials	RR and 95%CI	P value	I-square	P value for heterogeneity	RRR and 95%CI	Interaction P value
Publication year	2010 or after	8	0.60 (0.40–0.90)	0.014	70.1	0.001	0.79 (0.50–1.25)	0.311
	Previous 2010	11	0.76 (0.61–0.93)	0.009	17.6	0.276		
Country	Europe	9	0.89 (0.69–1.15)	0.381	21.9	0.248	1.53 (1.05–2.25)	0.029
	Other	10	0.58 (0.44–0.78)	<0.001	53.0	0.024		
Sample size	100 or greater	5	0.84 (0.63–1.12)	0.228	47.1	0.109	1.40 (0.93–2.11)	0.109
	<100	14	0.60 (0.45–0.81)	0.001	46.4	0.029		
Surgery sites	Thoracotomy or Laparotomy	17	0.73 (0.60–0.89)	0.002	45.0	0.023	2.70 (0.49–14.88)	0.253
	Other	2	0.27 (0.05–1.48)	0.133	45.6	0.175		
Follow-up duration (months)	12 or greater	8	0.63 (0.41–0.97)	0.037	36.8	0.135	0.89 (0.54–1.45)	0.634
	<12	11	0.71 (0.56–0.90)	0.005	56.6	0.011		

**Figure 3:**
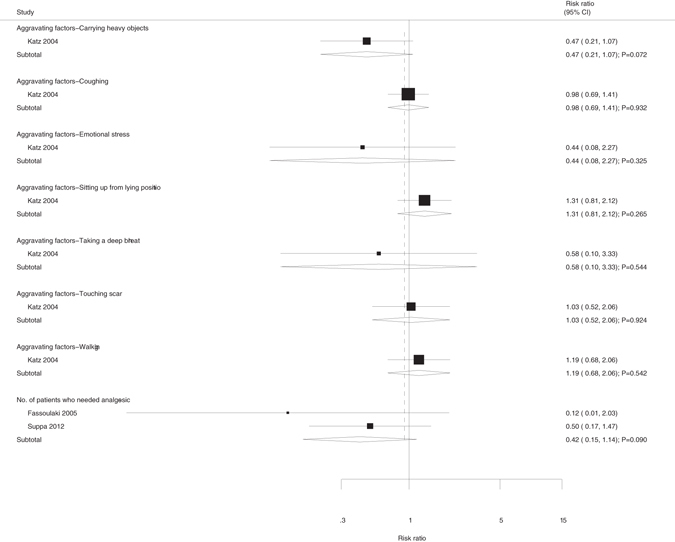
Summary of all bivariate outcomes.

In addition, other bivariate outcomes (Fig. 3) from aggravating factors causing pain, such as carrying heavy objects (RR, 0.47; 95%CI, 0.21–1.07; p = 0.072), coughing (RR, 0.98; 95%CI, 0.69–1.41; p = 0.932), emotional stress (RR, 0.44; 95%CI, 0.08–2.27; p = 0.325), sitting up from a prone position (RR, 1.31; 95%CI, 0.81–2.12; p = 0.265), taking a deep breath (RR, 0.58; 95%CI, 0.10–3.33; p = 0.544), touching the wound (RR, 1.03; 95%CI, 0.52–2.06; p = 0.924), and walking (RR, 1.19; 95%CI, 0.68–2.06; p = 0.542), showed no significant differences between the regional and no anesthesia groups. The above results were based mainly on the 2004 Katz study^18^ in which there was no statistical difference in the number of patients who needed analgesics regardless of whether regional anesthesia was used (RR, 0.42; 95%CI, 0.15–1.14; p = 0.090).

**Figure 4:**
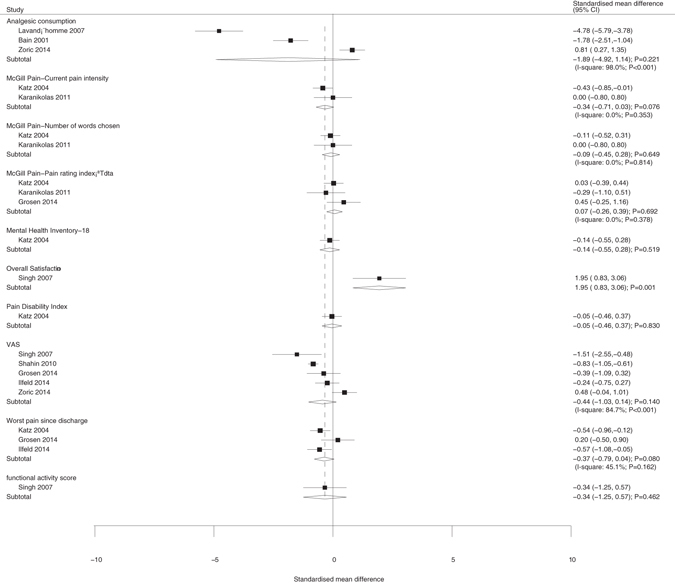
Summary of all continuous outcomes.

The overall satisfaction of the continuous variable outcomes (Fig. 4) showed significant differences between the groups (SMD, 1.95; 95%CI, 0.83–3.06; p = 0.001). Other outcomes, such as analgesic consumption (SMD, −1.89; 95%CI, −4.92–1.14; p = 0.221), current pain intensity based on the McGill index (SMD, −0.34; 95%CI, −0.71–0.03; p = 0.076), number of words chosen (SMD, −0.09; 95%CI, −0.45–0.28; p = 0.649), pain rating index (SMD, 0.07; 95%CI, −0.26–0.39; p = 0.692), mental health inventory-18 (SMD, −0.14; 95%CI, −0.55–0.28; p = 0.519), pain disability index (SMD, −0.05; 95%CI, −0.46–0.37; p = 0.830), VAS (SMD, −0.44; 95%CI, −1.03–0.14; p = 0.140), worst pain since discharge (SMD, −0.37; 95%CI, −0.79–0.04; p = 0.080), and functional activity score (SMD, −0.34; 95%CI, −1.25–0.57; p = 0.462), showed no significant differences between the regional and no anesthesia groups.

**Figure 5:**
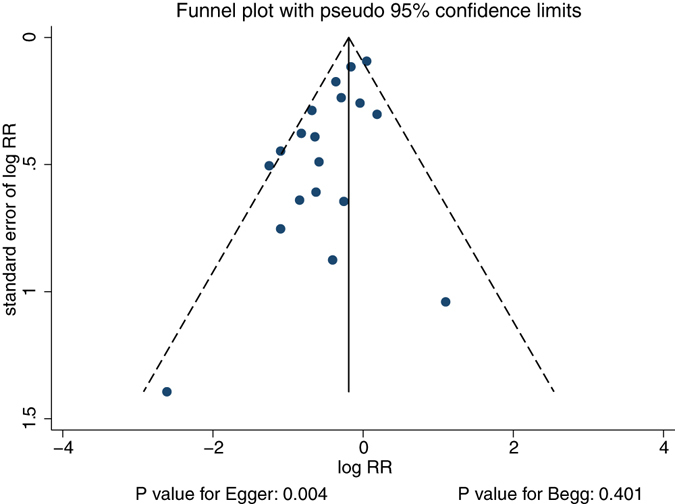
Funnel plot on chronic pain frequency.

A review of the funnel plots could not rule out the potential for publication bias of pain frequency (Fig. 5). Although the Begg’s test showed no evidence of publication bias for pain frequency (P = 0.401), a significant publication bias was detected using the Egger’s test (P = 0.004). The conclusions were not changed after adjustment for publication bias using the trim and fill method^39^.

## Discussion

Our meta-analysis was based on 21 RCTs, which included regional anesthesia for the prevention of chronic pain after major surgery. This large quantitative study included 1,980 patients with a broad range of populations. We found that regional anesthesia could significantly reduce postsurgery pain frequency and improve overall patient satisfaction. Subgroup analysis showed significant differences between regional and traditional anesthesia and was country specific. Regional anesthesia technology has been used extensively for multimodal anesthesia since it was developed; however, large numbers of studies supported the limitations on its effects and complications; therefore, researchers have begun to question the superiority of regional anesthesia on postsurgery pain. Epidural anesthesia has had an ideal postsurgery analgesic effect with less impacts on respiratory and gastrointestinal functions; however, during major surgeries, especially during joint replacement surgery, routine anticoagulants are used to prevent lower limb venous thrombosis, which would significantly increase the risk of iatrogenic epidural bleeding and limits epidural anesthesia application in major orthopedic surgeries. Others techniques, such as local nerve blocks, were widely accepted in surgeries and could reduce the amount of analgesic drugs with fewer complications; however, its area of action is less compared to that of epidural anesthesia and was frequently used in only minor orthopedic surgeries. In addition, the duration of this technique is relatively short, and whether it extends the blockade time or increases the frequency to improve the analgesic effect still needs further research. Local infiltration anesthesia blocks opioid receptors in the inflamed tissues to increase the overall effect of anesthesia; however, this method is used more in minor surgeries, such as breast surgery and hernia repair, and, at times, in major orthopedic surgeries as an additional and important component of multimodal anesthesia, but whether local infiltration anesthesia significantly prevents postsurgery pain must still be confirmed. Although regional anesthesia as part of multimodal anesthesia is gradually being accepted by researchers, controversies exist in clinical practice. Our study systematically analyzed whether the combined application of regional anesthesia could improve postsurgery pain, and the results indicated that regional anesthesia has significant advantages in postsurgery pain frequency and overall satisfaction; however, there was insufficient data for robust conclusions based on other study results.

Most of our secondary findings were in agreement with the trial conducted in Canada^18^, which comprised 83 patients who underwent major gynecological surgery by laparotomy. The results showed that although patients who received epidural analgesia had fewer disabilities 3.0 weeks after surgery, there was no significant impact on chronic pain frequency, which might have been because of their baseline and postsurgery measurements, such as psychological, emotional, and physical variables. Senturk *et al*.^24^, in their clinically, randomized, prospective study, suggested that patients who received thoracic epidural analgesia before the initiation of surgery showed no acute and long-term thoracotomy pain. In contrast, Shahin *et al*.^27^ indicated that intraperitoneal instillation decreased the incidence and scores of postcesarean pain after the parietal peritoneum was sutured. Strazisar *et al*.^33^ illustrated that wound infusion with a regional anesthetic reduced acute and chronic pain and opioid consumption, and also resulted in less postsurgery sedation and the need for antiemetics. Most of the trials showed no significant differences in chronic pain frequency, which might have been because of the trial design with acute pain control as the primary endpoint and the relatively small sample sizes, which did not allow the adequate statistical ability to detect potential clinical differences; therefore, large-scale RCTs should be conducted to verify the treatment effects on chronic pain.

Significant differences between Europe and other countries were observed for the effect of regional analgesia on chronic pain frequency. These results were somewhat surprising and the reasons remain unclear, but it is possible that the surgery sites and dosage of analgesics might play an important role in this difference. Most trials conducted in Europe included patients who underwent major surgeries, which might have required multimodal anesthesia to alleviate acute and chronic pain. The results of our data varied from that of other studies. Although fewer trials provided data about the pain scale, analgesia consumption, and adverse events, variable conclusions were reached.

## Conclusions

Regional analgesia significantly reduced the incidence in patients of chronic postsurgery pain at different surgery sites compared to that with traditional analgesia, but the analgesic efficacy of regional analgesia might not be similar in studies conducted in different countries. Future trials that focus on the long-term efficacy of regional analgesia in specific populations, including the characteristics of patients, are warranted.

### Study Limitations

Our meta-analysis had several limitations. First, the results were based on other studies, but not at the individual level. Second, there was relatively high heterogeneity in our analysis; therefore, the random-effects model was used to take possible heterogeneity into consideration. In addition, subgroup analyses were conducted based on the publication year, country, sample size, surgical sites, and follow-up duration to further explore the source of heterogeneity; however, unexplained heterogeneity also persisted, which might have been from the varying use of combined analgesia, different disease status, and surgical approaches. Third, the results of the data on most of the outcomes were too small to reach robust conclusions. Finally, the quality and reliability of our results might be limited by the quality of the underlying data. In the future, it is highly recommended that unified results for assessing the criteria are found, especially in small-sample studies.

## Materials and Methods

This meta-analysis was performed according to the Preferred Reporting Items for Systematic Reviews and Meta-Analyses (PRISMA) statement^40^.

### Search strategy and study selection

We conducted a literature search of PubMed, EmBase, and the Cochrane Central Register of Controlled Trials databases for articles published up to July 2016 using the following core search terms: “Anesthesia”, “Anesthetics”, “Analgesia”, “regional”, “local”, “Pain”, and “Postoperative”. The potential eligible studies with the titles and abstracts were reviewed to identify additional candidate studies, the reference lists of the included studies, and reviews.

Two authors independently extracted data from the literature search using a standardized approach. In the case of inconsistencies between these two authors, a consensus was reached by group discussion. The inclusion criteria were as follows: 1. randomized controlled trial studies; 2. studies focused on prevention and treatment of chronic postsurgery pain under different surgical categories; 3. study reports at least one outcome, such as pain frequency, pain scale, analgesic consumption, and adverse events; 4. studies published in English; and 5. a follow-up >30 d. The exclusion criteria were reviews, editorials, non-human studies, letters, and conference papers without sufficient data.

### Data collection and quality assessment

Two reviewers independently extracted data, and the disagreements were resolved by consensus with a third-party investigator. The following items were extracted from the included articles: author, country, sample size, patient status, regional technique, outcomes, and follow-up duration. A quality assessment was independently performed by two authors using an established tool, the Jadad scale, in the following five domains: randomization (1 or 0), concealment of treatment allocation (1 or 0), blinding (1 or 0), completeness of follow-up (1 or 0), and the use of intention-to-treat analysis (1 or 0)^41^. The studies were scored based on these results, which were then used to assess the methodological quality of clinical trials.

### Statistical analyses

A random-effects model was used to pool continuous data because of the high clinical heterogeneity among the studies and the results are presented as the standardized mean difference (SMD) and 95% confidence interval (CI) using the inverse variance method. Dichotomous data and results were summarized using risk ratio (RR) and 95% CIs using the Mantel-Haenszel method^42, 43^. Heterogeneity among studies was investigated using Q statistics, and P < 0.10 indicated significant heterogeneity^44, 45^. Subgroup analyses were conducted to assess the pain frequency on the basis of publication year, country, sample size, surgical sites, and follow-up duration. The relative risk ratios (RRRs) and the corresponding 95% CIs for pain frequency were estimated based on the publication year, country, sample size, surgical sites, and follow-up duration^46^. A sensitivity analysis was performed by removing each individual trial to evaluate the influence of each included study^47^. Egger^48^ and Begg’s^49^ tests were conducted and funnel plots created to check for any potential publication bias. All tests were two tailed, and p < 0.05 was considered statistically significant. We analyzed the data using STATA ver. 12.0 (StataCorp LLC, College Station, TX, USA).
